# The diagnostic utility of bone marrow examination in an infectious disease ward

**DOI:** 10.4102/sajhivmed.v20i1.974

**Published:** 2019-09-30

**Authors:** Nirvana Bharuthram, Charles Feldman

**Affiliations:** 1 Department of Internal Medicine, Faculty of Health Sciences, University of the Witwatersrand, Johannesburg, South Africa

**Keywords:** bone marrow examination, HIV, TB, infectious disease, internal medicine

## Abstract

**Background:**

Patients with advanced human immunodeficiency virus often present with unexplained fevers or cytopenias. Bone marrow aspirate and trephine examinations are an invasive means to aid diagnoses in patients who present with diagnostic dilemmas.

**Objectives:**

A retrospective record review to assess the diagnostic utility of bone marrow examinations in a South African Infectious Diseases ward.

**Methods:**

The records of patients who had undergone a bone marrow examination in the Infectious Disease ward at the Charlotte Maxeke Johannesburg Academic Hospital, Johannesburg, South Africa, between 01 January 2012 and 31 December 2014 were reviewed. A unique diagnosis was considered to be any diagnosis made on bone marrow examination alone, or a diagnosis made more timeously on bone marrow examination than with alternative investigations.

**Results:**

Of 327 patients who underwent bone marrow examination, 80 unique diagnoses were obtained in 77 cases (23.5%). The unique diagnoses included the presence of granuloma (*n* = 49), Mycobacterium tuberculosis (*n* = 17), Mycobacterium avium complex (*n* = 3), haematological malignancy (*n* = 4) and pure red cell aplasia (*n* = 5). A white cell count ≤ 4 × 10^9^/L predicted a unique outcome (*p* < 0.01). A white cell count ≤ 4 × 10^9^/L and CD4 cell count ≤ 50 cells/mm^3^ predicted mycobacterial infection of the bone marrow.

**Conclusions:**

The findings of a unique diagnosis in 23.5% of bone marrow examinations performed suggests that this remains a useful investigative modality in patients in whom less invasive investigations have not yielded a diagnosis.

## Introduction

South Africa (SA) has an immense burden of infection with human immunodeficiency virus (HIV). The 2016 data released by Statistics South Africa (SSA) estimated that 12.7% of the population was infected with HIV.^1^ Since 2005, there has been a decline in the annual death rate from the acquired immune deficiency syndrome (AIDS).^1^ Despite this, up to a third of HIV-infected patients still present with advanced disease and with low cluster of differentiation 4 (CD4) counts viz. CD4 < 100 cells/mm³.^2^ These individuals frequently present a diagnostic challenge to infectious disease (ID) physicians as their presentation is often associated with fever, non-specific symptomatology and cytopenias.^3,4^

A bone marrow aspirate and trephine examination is one of the modalities utilised when investigating such individuals. Since 1995 several local and international studies have evaluated the diagnostic utility of the bone marrow examination (BME), particularly in the setting of HIV. Diagnostic utility in this context refers to the number of diagnoses obtained from such a procedure. These studies have demonstrated a diagnostic utility in 25% – 47% of cases.^3,4,5,6,7,8,9,10^ Collectively, the most common positive finding is the presence of mycobacterial infection.^5,6,7,9,11,12,13,14^ There are two studies that have focused on the South African population: Karstaedt et al.,^3^ between 1996 and 1997, found that 38% of BMEs of HIV-seropositive individuals provided a diagnosis, of which 24% were ‘unique’, that is, diagnoses made on BME alone. These included Mycobacterium tuberculosis (MTB), Mycobacterium avium complex (MAC), disseminated cryptococcal infection and haematological malignancies. Bone marrow examinations performed in Cape Town in 2007 by Van Schalkwyk et al.^4^ yielded a diagnosis in 47% of 147 HIV-seropositive patients. Of these, 33% were unique diagnoses and included immune thrombocytopenic purpura (ITP), disseminated tuberculosis and haematological malignancies.

Previous SA studies of BME utility have confirmed MTB as the most frequent diagnosis. The last such study analysed data from 2004 to 2007. But since 2004, access to antiretroviral therapy (ART) has increased considerably.^15^ The diagnosis of MTB infection has similarly improved following the introduction of routine geneXpert MTB/RIF assessment of sputum and other body tissues and fluids in the clinics and hospitals of SA.^16^ What effect will these changes have on the future usefulness of BME in SA patients with suspected and difficult to diagnose infections?

## Methods

### Study design and setting

This was a retrospective record review of bone marrow aspirate and trephine examinations performed on adults admitted to the infectious disease (ID) ward of the Charlotte Maxeke Johannesburg Academic Hospital (CMJAH), in Johannesburg, SA, from 01 January 2012 to 31 December 2014.

### Study population

Any patients, irrespective of their suspected diagnosis and HIV status, who had a BME in the ID ward during the study period were included in the study. There were no exclusion criteria. Patients admitted to the ID ward included any patient aged ≥ 16 years with a suspected ID that may or may not have potentially required isolation during their hospital stay, examples of which were patients with suspected tuberculosis, meningitis and opportunistic infections related to HIV infection.

### Data collection and definitions

The results of the bone marrow aspirate and trephine studies were obtained from the National Health Laboratory Services’ (NHLS) database. Data were extracted from these reports and transferred to a standardised data collection sheet. Each record was allocated a number and identifying features were removed. Only laboratory tests performed at the time of or within a month before or after the BME were used for this analysis.

The parameters documented were the patients’ age, gender, HIV status, CD4 and viral load (VL) as well as pre-BME blood test results, namely the full blood count, reticulocyte production index, and vitamin B12, ferritin and folate levels. Cytopenias were recorded. The indications for the BME as well as the final clinical diagnoses were documented. The diagnosis of MTB on BME was made through a positive bone marrow culture and/or positive MTB polymerase chain reaction (PCR) test. At the time of the study direct PCR testing of bone marrow for MTB was not routine practice; where such data was available it has been included in the study. The diagnosis of MAC was made on a positive bone marrow culture. The presence of acid-fast bacilli on Ziehl–Neelsen (ZN) stain and/or that of granuloma on trephine examination suggested a likely mycobacterial infection. However, the microbiological diagnosis remained unconfirmed unless marrow culture or simultaneous tissue or fluid sampling elsewhere in the patient provided positive identification of the pathogen.

If relevant clinical information or data were not available on the NHLS database, the individual patient’s hospital records were obtained and reviewed. Sputum for geneXpert MTB/RIF was routinely sent on all ID ward patients with a productive cough. Where indicated, the following tests were also performed: lumbar puncture, pleural tap or fine needle aspirate. A BME was performed if these investigations failed to provide a diagnosis. The results of all investigations were obtained from the NHLS database.

For the purposes of this study, the term ‘unique diagnosis’ refers to any diagnosis made on the BME that was not made with any other diagnostic tests or if the BME provided an answer more timeously than alternative tests. In particular, the latter refers to bone marrow cultures that flagged positive before other specimens. These ‘unique’ results were important for patient care and may have influenced subsequent outcomes.

### Statistics

Statistica, version 13, and Stata were used to analyse the data. Descriptive statistics were used for patient demographics. Means and standard deviations were used for haematological parameters. The Student’s *t*-test was used for comparison between continuous data with a normal distribution. The Mann–Whitney-U test was used in comparisons between data without a normal distribution, specifically in the comparison between possible predictive variables and unique diagnoses. A statistically significant result was defined as a *p*-value of < 0.05. Odds ratios (OR) were calculated for predictive determinants of a unique diagnosis on BME.

### Ethical consideration

Ethics approval was obtained from the University of the Witwatersrand Human Research Ethics Committee (clearance number M150847).

## Results

### Baseline characteristics

A total of 327 bone marrow aspirate and trephine examinations were carried out in the adult ID ward during the study period. The study population consisted of 162 (49.5%) males and 165 (50.5%) females. The mean age of the study population was 36 years with a range of 17–65 years.

Overall, 314 patients (96%) were HIV-seropositive and 12 HIV-seronegative (3.7%). One patient’s HIV status was unknown. Amongst those with HIV infection, the median CD4 cell count was 47 cells/mm^3^ (1 cells/mm^3^ – 1069 cells/mm^3^) and 271 patients (86.3%) had a CD4 cell count of ≤ 200 cells/mm^3^. There were 128 patients on ART (40.8%) at the time of the bone marrow investigation.

The major indication for performing a BME was for the investigation of a peripheral blood cytopenia (54% of cases). Thirty-two per cent of BMEs were performed due to a clinical suspicion of MTB and 9% were performed owing to a suspicion of a malignant lesion with bone marrow involvement. Other indications, accounting for 5% of BMEs performed, were for the investigation of a thrombocytosis, leucocytosis or suspected disseminated cryptococcal infection.

### Unique diagnoses made on bone marrow examination

Of the 327 BMEs performed, 158 (48.3%) yielded positive results (Table 1). Three of these cases yielded two diagnoses each. The most common findings were the presence of granulomata on trephine examination (57 cases, 17.4%) and a positive bone marrow culture for MTB (50 cases, 15.3%). The presence of granulomata together with a positive ZN stain on BME was found in 32 cases (9.8%).

**TABLE 1:** All diagnoses on bone marrow examination.

Diagnosis	Number of patients	Percentage of study population (%)
**Microbiologically confirmed MTB**		
MTB culture positive on BME	48	14.68
MTB culture positive on BME (Rifampicin resistant)	1	0.31
MTB culture positive and TB PCR positive	1	0.31
TB PCR positive alone	5	1.53
Total	55	16.82
**Microbiologically confirmed MAC**		
MAC cultured on BME	4	1.22
Total	4	1.22
**Microbiologically proven mycobacterial infection**		
BME mycobacterial culture positive, organism unknown	1	0.31
Total	1	0.31
**Undifferentiated mycobacterial infection**		
Granulomata observed & ZN positive	32	9.79
ZN positive only	2	0.61
Total	34	10.4
**Possible mycobacteral infection**		
Granulomata observed only	57	17.43
Total	57	17.43
**Haematological malignancies**		
Non-Hodgkin’s lymphoma	2	0.61
Hodgkin’s lymphoma	1	0.31
Peripheral T cell lymphoma	1	0.31
Total	4	1.22
**Pure red cell aplasia**		
Parvovirus B19 PCR positive	2	0.61
Suspected Parvovirus B19 infection but PCR negative	1	0.31
Secondary PRCA, Parvovirus PCR negative	2	0.61
Total	5	1.53
**Other diagnoses**		
Aplastic anaemia	1	0.31
PAS stain positive, cryptococcus on BME	1	0.31
Total	2	0.61
**Total**	**158**	**-**

MTB, Mycobacterium tuberculosis; ZN, Ziehl-Neelsen; BME, bone marrow examination; MAC, Mycobacterium avium complex; MTBR, Mycobacterium tuberculosis rifampicin resistant; PCR, polymerase chain reaction; PRCA, pure red cell aplasia; PAS, Periodic acid-schiff.

In 77 cases (23.5% of the study population), the diagnoses made were unique to the BME (Table 2). Confirmed MTB was the unique diagnosis obtained in 17 of the 77 cases (22.1%) with unique diagnoses and confirmed MAC comprising three of the unique diagnoses obtained (2.6%). In two of these cases, MAC was diagnosed considerably faster on BME than peripheral blood culture (4 vs. 38 days and 8 vs. 26 days, respectively). Five patients had the diagnosis of a pure red cell aplasia made on BME. Four cases of haematological malignancies were identified. These comprised two cases of non-Hodgkin’s lymphoma (NHL), one case of Hodgkin’s lymphoma and one case of peripheral T cell lymphoma. Aplastic anaemia and disseminated cryptococcal disease were two additional unique diagnoses made on BME.

**TABLE 2:** Unique diagnoses on bone marrow examination.

Mycobacterial infection	Number of patients	Number of patients on TB treatment already
**Microbiologically confirmed MTB**		
MTB culture positive on BME only	9	3
BME MTB culture positive and granulomata observed	6	1
BME MTB culture positive and ZN positive and granulomata observed	2	1
**Microbiologically confirmed MAC**		
MAC cultured on BME only	1	1
MAC cultured on BME faster than on peripheral blood	2	0
Total	20	6
**Undifferentiated or possible mycobacterial infection**		
ZN positive with/without granuloma	12	5
Granulomata observed only	37	24
Total	49	29
**Pure red cell aplasia**		
Parvovirus B19 PCR positive	2	-
Suspected Parvovirus B19 infection but PCR negative	1	-
Secondary PRCA, Parvovirus PCR negative	2	-
Total	5	-
**Malignancies**		
Non-Hodgkin’s lymphoma	2	-
Hodgkin’s lymphoma	1	-
Peripheral T cell lymphoma	1	-
Total	4	-
**Others**		
PAS stain positive, cryptococcus on BME	1	-
Aplastic anaemia	1	-
Total	2	-

MTB, Mycobacterium tuberculosis; ZN, Ziehl-Neelsen; BME, bone marrow examination; MAC, Mycobacterium avium complex; MTBR, Mycobacterium tuberculosis rifampicin resistant; PRCA, pure red cell aplasia; PAS, Periodic acid-schiff.

Three patients had two unique diagnoses obtained on bone marrow investigation. The patient with Hodgkin’s lymphoma also had the finding of granulomata on BME and was on empiric TB treatment. One patient with a positive Parvovirus B19 PCR slide also had a positive TB bone marrow culture. This patient was also on empiric TB treatment at the time of the BME. The third patient, who was not on TB treatment at the time of the BME, had both the diagnosis of NHL as well as a positive ZN stain together with granulomata observed on BME.

Of the laboratory parameters measured, a white cell count (WCC) ≤ 4 × 10^9^/L was found to be a significant predictor of a unique diagnosis on BME (OR: 2.38 and 95% confidence interval [CI] 1.37–4.14) (*p* = 0.002) (Table 3). A neutrophil count ≤ 0.5 × 10^9^/L was found more commonly in those with a unique diagnosis (*p* = 0.05); however, the numbers in this group were too small to interpret correctly.

**TABLE 3:** Predictors of a unique diagnosis on bone marrow examination.

Variable	Unique diagnosis (*n* = 77)		Not a unique diagnosis (*n* = 250)		*p*	Odds ratio	95% CI
	*n*	%	*n*	%			
HIV positive	75	97.4	239	95.6	0.57	1.56	0.33–7.32
Male sex	40	51.9	122	48.8	0.65	1.12	0.67–1.88
Hb (≤ 7 g/dL)	32	41.6	95	38.0	0.58	1.16	0.69–1.95
Platelet count (≤ 150 × 10^9^/L)	52	67.5	177	70.8	0.58	0.86	0.50–1.49
WCC (≤ 4 × 10^9^/L)	55	71.4	128	51.2	0.002	2.38	1.37–4.14
Neutrophil count (≤ 0.5 × 10^9^/L)	5	6.5	5	2.0	0.05	3.55	1.00–12.67
CD4 (≤ 50 cells/mm^3^)	41	53.2	121	48.4	0.62	1.13	0.67– 1.92

WCC, white cell count; Hb, haemoglobin; CD4, cluster of differentiation 4; CI, confidence interval.

### Diagnosis of mycobacterial infection on bone marrow examination

Of the 327 bone marrow investigations performed, microbiological confirmation of MTB and MAC were made in 54 and 4 cases, respectively. Of the 226 bone marrow mycobacterial cultures performed 55 yielded positive results (24.3%). Of the 182 ZN stains performed 51 yielded positive results (28.0%). Six MTB PCRs were commented on as positive; however, it was not noted how many MTB PCR tests were performed that yielded negative results, and thus comparisons could not be performed. A WCC ≤ 4 × 10^9^/L and a CD4 cell count ≤ 50 cells/mm^3^ were found to be significant predictors of a microbiologically confirmed diagnosis of a mycobacterial infection (including MTB and MAC) on BME (Table 4).

**TABLE 4:** Predictors of mycobacterial infection on bone marrow examination.

Variable	Mycobacterial infection proven (*n* = 60)		Not proven mycobacterial infection (*n* = 267)		*p*	Odds ratio	95% CI
	*n*	%	*n*	%			
Hb (≤ 7 g/dL)	24	40.0	106	39.7	0.97	1.01	0.57–1.61
Platelet count (≤ 150 x 10^9^/L)	43	71.7	186	69.7	0.76	1.10	0.59–2.05
WCC (≤ 4 x 10^9^/L)	42	70.0	140	52.4	0.01	2.11	1.15–3.87
CD4 (≤ 50 cells/mm^3^)	40	66.8	124	46.4	0.005	2.37	1.28–4.41

WCC, white cell count; Hb, haemoglobin; CD4, cluster of differentiation 4; CI, confidence interval.

There were seven patients with the diagnosis of MAC in the study population. In three of these patients, the BME was not diagnostic and MAC was found on a positive peripheral blood culture alone. Of the remaining four patients with MAC in the study population, one cultured MAC on bone marrow culture alone, namely a diagnosis unique to the BME. Amongst the other three patients, both the bone marrow and peripheral blood cultures were positive for MAC. In two of these cases, MAC was cultured faster on bone marrow than peripheral blood culture (unique diagnoses) and in one case MAC was cultured faster on peripheral blood hence not a unique diagnosis. This resulted in three of the four cases of MAC diagnosed on bone marrow culture that were unique to the BME. In all the cases of MAC, the CD4 cell counts were ≤ 35 cells/mm^3^. Four of the five patients who were on ART at the time had virological failure and one of the five presented as a suspected immune reconstitution syndrome after having been on ART for 3 months.

## Discussion

In the current study, the cohort comprised patients from an ID ward at a tertiary level referral hospital covering a population of over 4 million individuals from central and northern Johannesburg.^17^ A total of 327 bone marrow investigations were performed during the 3-year study period. This sample size is larger per time period compared to international studies which, on average, have taken longer to review similar numbers viz. 8 to 9 years.^5,6,11^ This difference may be accounted for by the high burden of HIV infection and tuberculosis in this region compared to developed countries.^18^

The mean age of the study population was 36 years (17–65 years), with 96% of these individuals being HIV-seropositive. This reflects the high burden of HIV infection in the South African public sector.^15^ The median CD4 cell count at admission was 47 cells/mm^3^ (1 cells/mm^3^ – 1069 cells/mm^3^) reflecting not only delayed health-seeking behaviour but also the higher morbidity in those with advanced stages of HIV infection and CD4 cell counts < 200 cells/mm.^3,19^ Such patients with lower CD4 cell counts are more likely to have clinically advanced disease and a lower anticipated survival rate. At the time of the BME, 40.8% of patients were already on ART. The low CD4 counts noted on presentation to a hospital despite being on ART suggest that these individuals were either being treated unsuccessfully or that their ART had been initiated at a late stage in their clinical decline. The number of patients on ART at the time of this study is higher than the 23% of patients on ART found in the study by Van Schalkwyk et al.^4^ between 2004 and 2007. This difference can be accounted for by the expanded rollout of ART from 2004 onwards.^20^ Only *n* = 12 (4%) of the cohort was HIV-uninfected. This small number did not permit meaningful comparisons between the HIV-seronegative and HIV-seropositive groups.

### Unique diagnosis on bone marrow examination

Positive results on BME were found in 48.3% of cases, which highlights the important persistent utility of BME in the investigation of patients with advanced immunosuppression and unexplained cytopenias, fever or the suspicion of disseminated mycobacterial infection. The finding of positive results in 48.3% of cases was marginally higher than previous studies in which diagnoses made on BME accounted for 25% – 47% of cases.^3,4,5,8,9,10,12,13,14,21^ In the current study, 23.6% of diagnoses made were unique to the BME. This finding was similar to the 24.9% unique diagnoses on BME found by Karstaedt et al.^3^; furthermore, this is also higher than international studies in which the prevalence of unique diagnoses has ranged between 8% and 10%.^3,13^ Van Schalkyk et al.,^4^ however, had an even higher yield of 33% unique diagnoses in their cohort of 147 patients. The higher yield of unique diagnoses across South African studies may reflect the fact that in the developing world because of resource constraints this investigative modality is not widely available and, therefore, more consideration is given prior to performing a BME, thus those investigations that are performed are more likely to yield a positive result. Despite this, the study’s findings may be important to those practitioners in resource-constrained areas of SA as a guide as to which patients are likely to benefit from BME to aid with diagnosis.

The predominant unique diagnosis made on BME in the current study was that of MTB, which encompassed 22.1% (17/77) of BMEs with a unique diagnosis. This finding is in agreement with the findings by both Van Schalkwyk et al.^4^ and Karstaedt et al.,^3^ which also revealed MTB as the predominant unique diagnosis on BME. Brooke et al.^5^ in 1997 reviewed 215 bone marrow samples over a 9-year period in London and similarly found MTB as the predominant diagnosis (20% of all cases).

Other unique diagnoses found in the current study, which were similarly found in other studies, were disseminated MAC, haematological malignancies and disseminated cryptococcosis.^11,14,21^ Most international studies, however, found the prevalence of MAC to be higher than that of MTB, reflecting differing disease profiles of mycobacterial infection in developing countries like SA in which MTB is predominant versus the developed countries in which MAC appears to be a more common diagnosis on BME.^22^

### Predictors of a positive bone marrow examination result

This study found that a WCC ≤ 4 × 10^9^/L was a positive predictor of a unique diagnosis on BME (*p* = 0.002). A neutrophil count of ≤ 0.5 × 10^9^/L, although found more commonly in those with a unique diagnosis, was not found to be significant. Karstaedt et al.,^3^ similar to our study, found that a WCC < 4 × 10^9^/L predicted a unique diagnosis. Van Schalkwyk et al.^4^ found that a neutrophil count < 0.5 × 10^9^/L predicted a unique BME result; however, that study additionally found that a Hb < 6 g/dL was also a significant predictor of a unique diagnosis. More advanced HIV disease, with a lower CD4 cell count, was found to be an additional predictor of a positive BME in some international studies.^3,13^ Keiser et al.^13^ and Luther et al.^6^ both found that a haematocrit < 25% and 30%, respectively, predicted positive results. This variable; however, was not measured in the current study.

### Mycobacterial infection on bone marrow examination

Just over one-fifth of the unique diagnosis observed on BME resulted from confirmed MTB infection (22.1%). This finding highlights the persistent utility of the BMEs in diagnosing MTB despite the introduction of the Xpert MTB/RIF testing in early 2011.^16^ GeneXpert MTB/RIF testing of sputum has a reported sensitivity of 88% and a specificity of 99%.^23^ Between 2012 and 2014, 690 435 Xpert MTB/RIF tests had been performed in Gauteng with the diagnosis of MTB made in 12% of cases.^16^ Despite access to this useful investigative modality being available at the CMJAH during the study period, we demonstrate that the BME still had utility to not only aid with diagnosis of MTB but also provide additional diagnoses that may not have been clinically suspected such as malignancies. An additional consideration is that many ill patients are unable to provide good quality sputum samples. Although induction of sputum may be attempted if this is unsuccessful, the availability of alternative diagnostic means such as BME is necessary.^24^ In recent years, the urine lipoarabinomanin (urine LAM) test has become available as another modality to aid diagnosis of disseminated MTB infection; however, this testing modality was not available at our centre at the time of the study and its influence on the future necessity of BME is still to be determined.^25^

#### Granulomata on bone marrow examination

In 57 cases granulomata were the only positive finding on the BME. Although there are potentially multiple causes of granuloma on BME, in the setting of advanced HIV and with a clinical suspicion of MTB in 32% of cases, the presence of granuloma in the clinical setting would add value in assisting with the decision to possibly initiate a patient on empiric MTB treatment. Lin et al.,^12^ in their study based in Taiwan, found that patients with MTB infection were more likely to have granulomata on BME than those with non-tuberculosis mycobacterial infections (82% compared to 30% of their 24 patients; *p* = 0.03). This finding together with the high prevalence of HIV positive patients with lower CD4 cell counts possibly accounts for the higher prevalence of granulomata observed on BME in South African studies. Riley et al.^11^ found that there were no positive MTB stains on BME in the absence of granulomata leading to their suggestion that the ZN stain is of no use in the absence of granulomata. A similar finding was observed in our study with 48 patients having both a positive ZN stain and granulomata observed on BME as compared to three patients with a positive ZN stain and no granulomata observed on BME. Despite this difference, the presence of a concurrent positive ZN stain increases the index of suspicion that the granuloma is associated with underlying mycobacterial infection rather than another cause.

#### Ziehl–Neelsen stain on bone marrow examination

Ziehl–Neelsen testing yielded positive results in 28% of cases. Van Schalkwyk et al.^4^ and Chosamata et al.^26^ found that 44% and 65%, respectively, of patients with microbiological proven diagnosis of MTB on BME had concurrent positive ZN stains. This finding is far more than the 29% (16 of 55 cases) found in the current study. This may be accounted for by the greater percentage of patients already on empiric tuberculosis treatment in the current study, with 43.6% (17 of 39 cases) of those with positive MTB cultures and negative ZN stains being on empiric treatment at the time of the study.

#### Predictors of Mycobacterium tuberculosis on bone marrow examination

This study found a WCC ≤ 4 × 10^9^/L and CD4 cell count ≤ 50 cells/mm^3^ were predictors of a confirmed diagnosis of mycobacterial infection on BME. A similar finding of a lower CD4 count in those with positive mycobacterial findings on BME was found in the 2015 study by Sedick et al.,^27^ in which the median CD4 cell count range amongst the different methods of diagnosing mycobacterial infection on BME was between 7 cells/mm^3^ and 33 cells/mm^3^. The statistical significance of this finding was, however, not evaluated.

#### Mycobacterium avium complex on bone marrow examination

Four individuals in this study had MAC cultured on BME, as compared to 49 who had MTB cultured. This finding is in contrast to studies conducted in the developed world, which reflect a higher prevalence of MAC infection as compared to MTB.^5,6,9,10,11,12,13^ In the London study by Riley et al.,^11^ MAC was the organism most commonly found on bone marrow culture (42 of 51 cases). The investigators also found that of the patients in their study diagnosed with MAC, this was a unique diagnosis in one third of the cases, which for them highlighted the usefulness of BME in aiding with MAC diagnosis. The utility of BME in diagnosing MAC in the current study was difficult to comment on given the small sample of patients who cultured MAC, namely 4 out of 55 positive bone marrow culture results. This finding may also reflect a sampling bias as MAC is difficult to diagnose and is often only looked for on BME if the clinical suspicion exists. Given the available literature, however, BME appears to be a useful means to diagnose MAC in areas with higher disease prevalence.

### Potential limitations of the study

There were a few potential limitations of this study. This was a single centre study undertaken in a single unit and so the results may not be generalisable to other units or to other hospitals in SA. Study patients were already in an ID ward with clinically advanced HIV at the time of the study, this at the outset would favour the presence of opportunistic infections, particularly MTB. Bone marrow findings were obtained from the retrospective review of published reports and individual bone marrow aspirate and trephine specimens were not reanalysed; we were, therefore, unable to control for any unidentified variables that may have influenced patient presentation. The subjectivity associated with the interpretation of the BMEs by the different assessors has to be noted. In addition, the fact that the study cohort was obtained specifically from an ID ward in which the yield of tuberculosis and the HIV sero-prevalence is anticipated to be higher than that of a general medical ward having patients with both communicable and non-communicable diseases may influence how the study findings may be applied to primary or district hospital settings in SA.

## Conclusion

The findings in this study conclude that bone marrow aspirate and trephine examinations still provide utility as a diagnostic tool even in the current South African setting of more comprehensive rollout of ART as well as increased availability of novel diagnostic tests for MTB. This finding applies particularly to patients in an ID ward in whom non-invasive standard investigations for the presence of cytopenias on peripheral blood have been negative and the concern of disseminated mycobacterial infection remains high. Performance of a bone marrow aspirate and trephine in these circumstances, through aiding with diagnosis, allows initiation and/or changes in patient treatment plans and by inference would most likely improve patient outcomes. The ongoing advancements occurring in the field of molecular diagnostics may in the future further revolutionise the efficacy and means of diagnostic tests utilised in such patients. Although the current study shows persistent diagnostic utility of BMEs, these ongoing advancements such as introduction of geneXpert Ultra testing may reduce the need for BME in the future. Furthermore, similar studies should be carried out and the current study would be a good marker to compare future studies against.

Our final recommendation would be to conduct the least invasive investigations first. If no success is obtained in making a diagnosis, a BME is then recommended. Mycobacterial cultures on blood and bone marrow specimens should be performed together to allow for optimal yield. Bone marrow trephine results should ideally be made available to the clinician within 2 weeks of the performance of the test. Unfortunately, this is often difficult in the resource-limited public sector in SA. It is imperative in those patients who have been started on empiric TB therapy to follow up the results of these invasive investigations in order to confirm the diagnosis and to exclude other unexpected diagnoses.
